# Supplementary material for: Identification and Verification of Biomarkers and Immune Infiltration in Obesity-Related Atrial Fibrillation
Source: Biology (Basel). 2023 Jan 12;12(1):121. doi: 10.3390/biology12010121 (PMC9855995; doi:10.3390/biology12010121)
Supplement: Supplementary file 1 [file biology-12-00121-s001.zip › Tables S1 and S2, Figures S1 and S2.pdf]

Table S1. Patients' baseline characteristics

	Lean-SR	Obese-SR	Lean-AF	Obese-AF
Number	7	8	8	8
Male n (%)	5 (71.4)	6 (75)	4 (50)	6 (75)
Age (in years)	38.8±19.2	58.7±7.1	73.4±13.3	59.5±9.6
History of AF (in months)	0	0	67.4±91.7	50.9±57.8
BMI (kg/m^2)	21.6±2.1	31.2±0.6	20.3±2.4	31.1±0.9
Diabetes n (%)	0 (0)	4 (50)	0 (0)	5 (62.5)
Hyperlipidemia n (%)	0 (0)	7 (87.5)	0 (0)	6 (75)
Hypertension n (%)	2 (28.5)	6 (75)	3(37.5)	7 (87.5)

Values are mean ± SEM or numbers (percentage).

Table S2. Primers used in this study for RT-qPCR experiments

Gene	Forward (5' to 3')	Reverse (5' to 3')
Homo MNDA	TGTCCCAAGAGCAGAGTAAGC	ACCACTGTCACTGGGTCGTT
Homo CYBB	AGGAGTTTCAAGATGCGTGGA	AGCTTCAGATTGGTGGCGTTA
Homo CD86	TGACCTGCTCATCTATACACGG	GCTGATGGAAACGTCGTACA
Homo FCGR2C	AAATGGAGACTGGAAAAATTCCTGA	CCCGTGAAGTTTTGCTTGTGT
Homo NCF2	CTCTCCGCAGTCCGACATC	AGCTTCACTTCCTTAGGCTCTTC
Homo LCP2	CCACCCTCCAATGACGAGG	GGGCGGCCATCGGTCT
Homo TLR8	CCAAACTGCCAAGCTCCCTAC	CCTCGGACAGTTCCCGCTTA
Homo HLA-DRA	GGCGGCTTGAAGAATTTGGAC	CACAGGGCTGTTTGTGAGCA
Homo LCP1	GAGAAGAGCGGACATTTAGGAA	GGGGTATGGCGGTTTGTTTA
Homo PTPN22	TCCTACAACTGTGGCTGAGAAG	TAGCTGGAATCCTCATCAGAGGT
Homo ACTB	GTTGCGTTACACCCTTTCTTG	GGGGATGCTCGCTCCAA

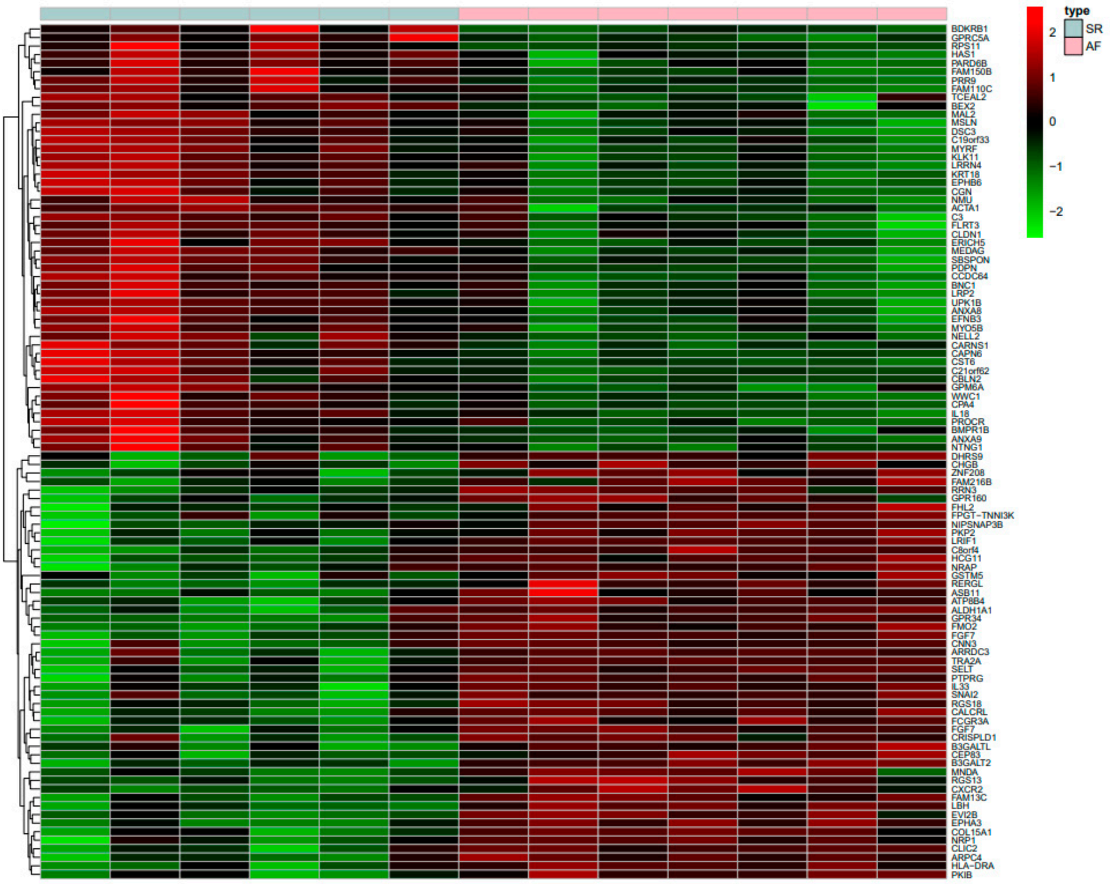

Figure S1. Hierarchical clustering analysis of AF-related top 50 up-regulated and top 50 down-regulated expressed genes.

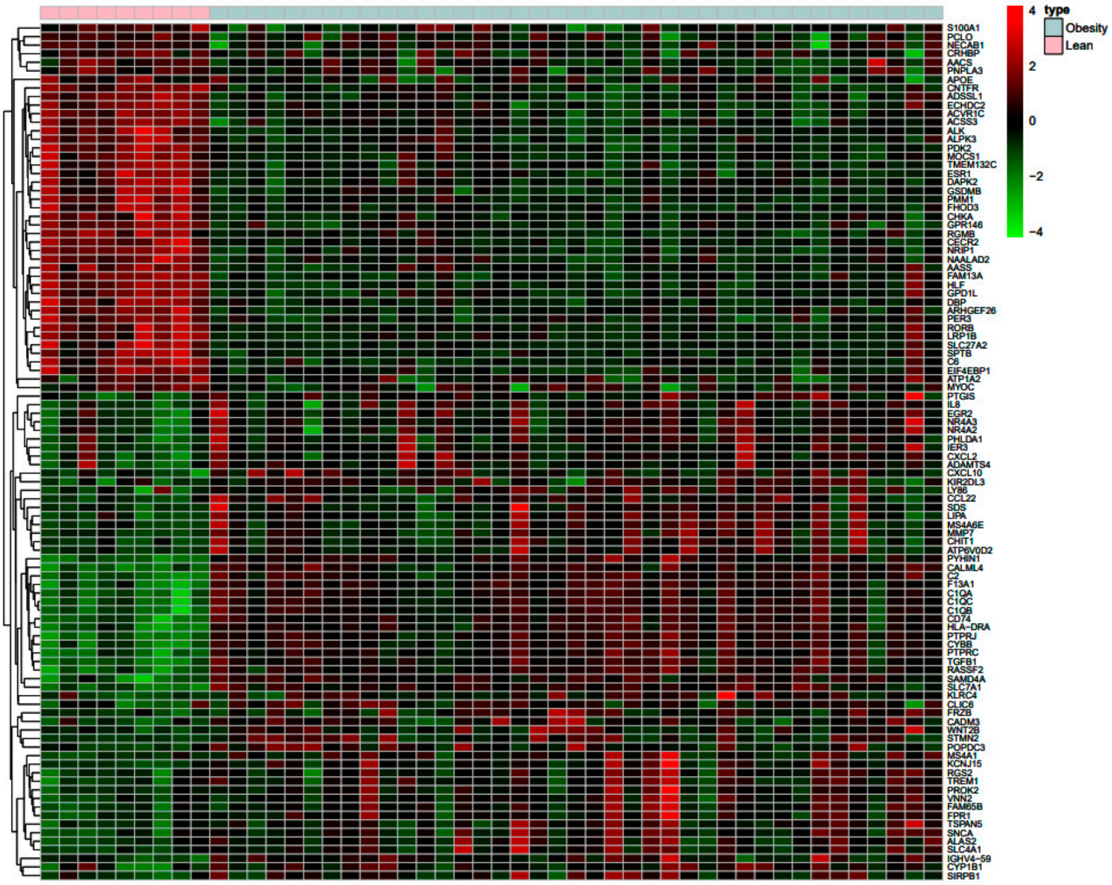

Figure S2. Hierarchical clustering analysis of obese-related top 50 up-regulated and top 50 down-regulated expressed genes.
